# Supplementary material for: Magnetically brightened dark electron-phonon bound states in a van der Waals antiferromagnet
Source: Nat Commun. 2022 Jan 10;13:98. doi: 10.1038/s41467-021-27741-3 (PMC8748959; doi:10.1038/s41467-021-27741-3)
Supplement: Supplementary file 1 — Supplementary Information [file 41467_2021_27741_MOESM1_ESM.pdf]

Supplementary Information for **Magnetically brightened dark electron-phonon bound states in a van der Waals antiferromagnet**

Emre Ergeçen[1,*], Batyr Ilyas[1,*], Dan Mao[1], Hoi Chun Po[1,2], Mehmet Burak Yilmaz[1], Junghyun Kim[3,4], Je-Geun Park[3,4], T. Senthil[1], and Nuh Gedik[1,†]

[1]Department of Physics, Massachusetts Institute of Technology, Cambridge, 02139, Massachusetts, USA.
[2]Department of Physics, Hong Kong Univesity of Science and Technology, Clear Water Bay, Hong Kong, 999077, China.
[3]Center for Quantum Materials, Seoul National University, Seoul 08826, Republic of Korea.
[4]Department of Physics and Astronomy and Institute of Applied Physics, Seoul National University, Seoul 08826, Republic of Korea.
[†]e-mail: gedik@mit.edu
[*]These authors contributed equally to this work.

1 Experimental Setup

Below we show schematics of two experimental setups used in this work. In Figure S1a, broadband transient absorption spectroscopy setup is shown. Yb-based regenerative amplifier output is splitted into two arms. First arm seeds optical parametric amplifier (OPA) and wavelength tunable output of it used as a pump. Electro-optic modulator on the pump path chops pulse trains at high frequency (50 kHz). The second arm with power tuning optics (half-waveplate and beam splitter), is focused on calcium fluoride (CaF_2) crystal to generate white-light continuum. White-light pulses with a stage to control delay time from pump pulses, were used to measure transient absorption changes in $NiPS_3$. Both beams were focused onto sample surface with a lens and reflected probe beam was sent to a photodiode (PD) through monochromator.

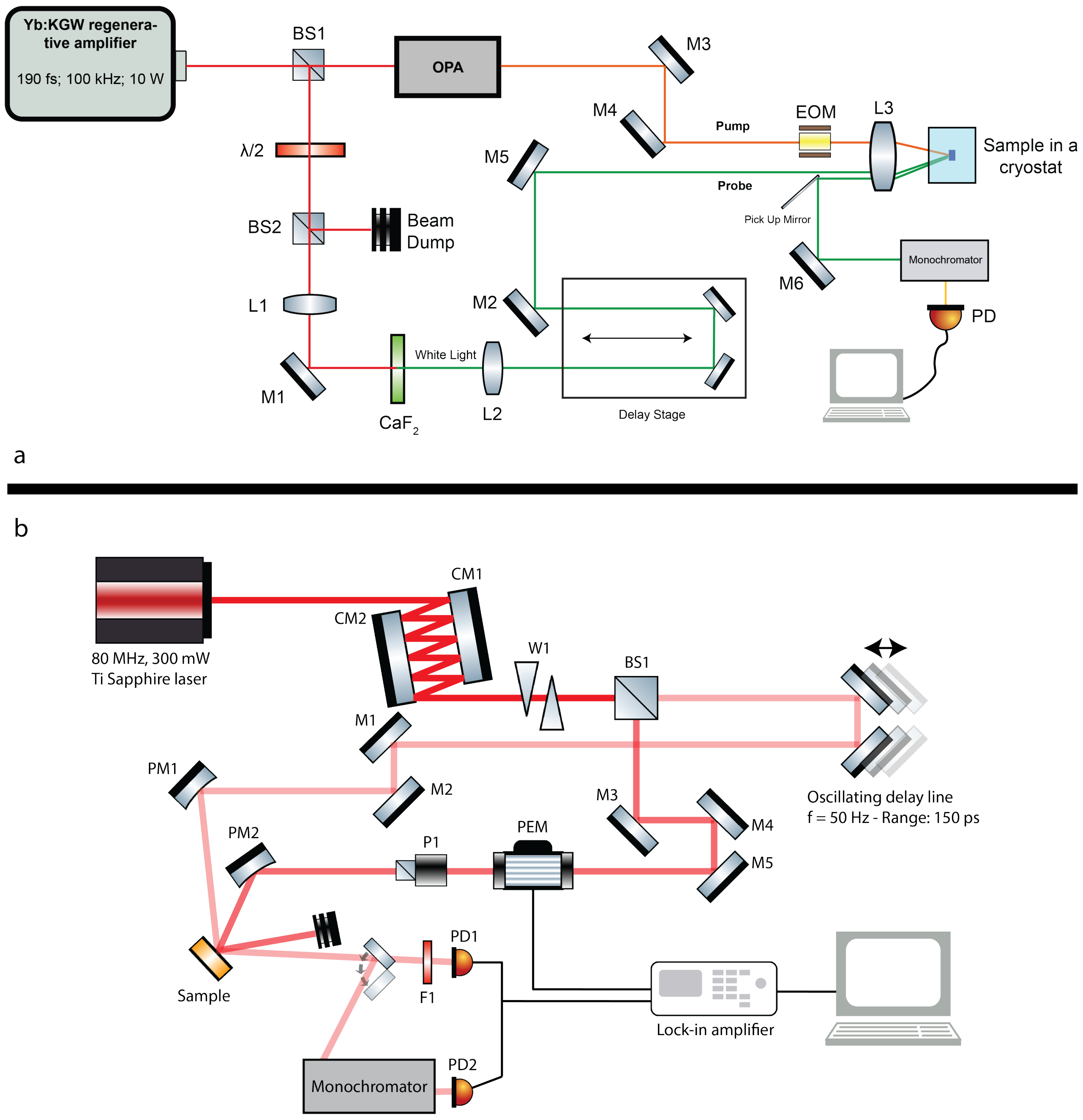

Figure S1: Schemes of two experimental setups: **a.** Broadband transient absorption spectroscopy; **b.** Energy resolved coherent phonon spectroscopy. "BS" - Beam Splitter; "$\lambda/2$" - Half Waveplate; "M" - Mirror; "PM" - Parabolic Mirror; "CM" - Chirped Mirror; "W" - Wedge; "P" - Polarizer; "PD" - Photodiode; "L" - Lens; "EOM" - Electro-Optics Modulator; "PEM" - Photoelastic Modulator;

In Figure S1b, we show energy resolved coherent phonon spectroscopy setup. Ti:Sapphire oscillator output sent to a pair of chirped mirrors (CM) and wedges (W), to precompensate for group velocity dispersion (GVD) caused by all optical elements in the setup. This ensures us to maintain short pulse durations (∼25 fs) down to sample position. After that the beam was split into pump and probe arms. We used a photo-elastic modulator (PEM) to chop pump beam at 100 kHz. Probe beam, reflected from sample, is sent to photodiode (PD) either through a long-pass filter (F1) or a monochromator. A fast oscillating mirror on probe arms allowed us to collect more signal statistics and increase signal-to-noise ratio.

To coherently launch a phonon mode, the excitation pulse must have a pulse duration shorter than the phonon period. To fulfill this requirement, we used a broadband ultrafast laser source (Cascade-5, KMLabs). Pulse durations at the sample position are characterized by a frequency resolved optical gating (FROG) setup. The autocorrelation trace is overlaid on the FROG trace is given in Figure S2. Full width half maximum (FWHM) of the autocorrelation trace is equal to 42.5 fs. To extract the pulse duration, we divided the FWHM value by a factor of 1.53, assuming a Gaussian pulse shape and estimate our pulse duration as 27.76 fs.

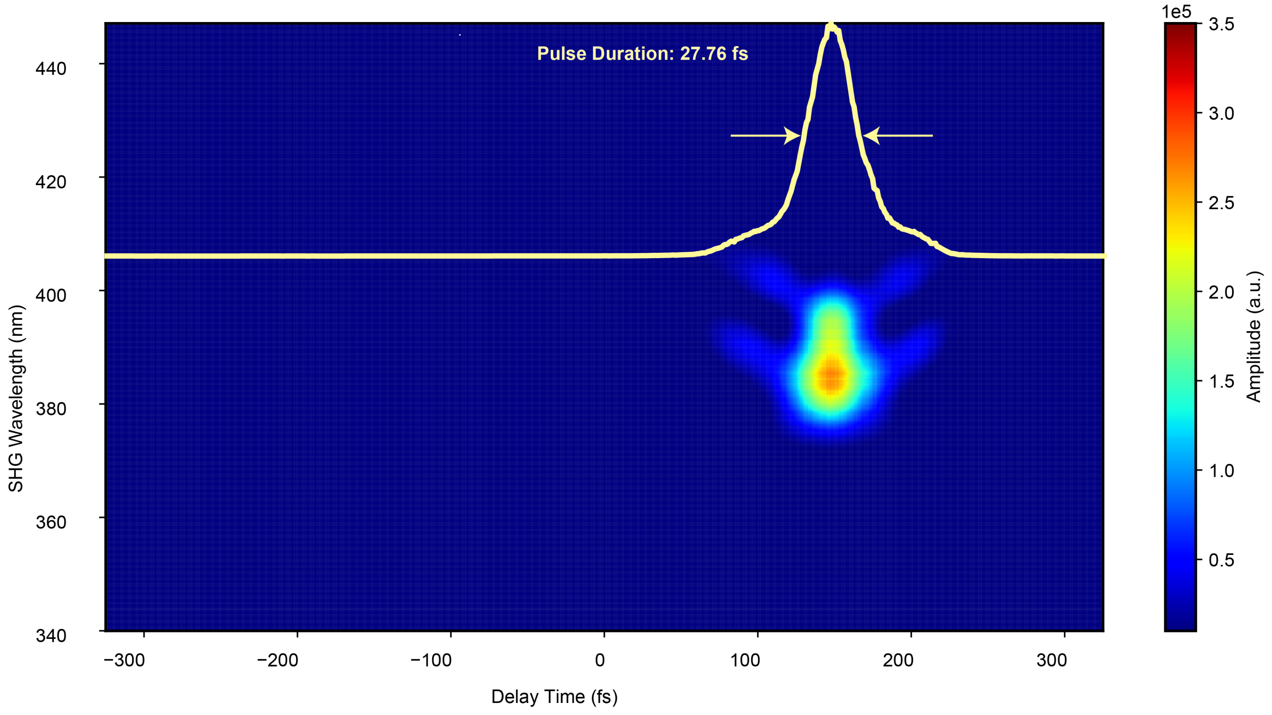

Figure S2: Pulse duration characterization of a energy resolved coherent phonon spectroscopy setup, using FROG technique. Line plot in yellow is obtained by integrating over frequencies and was fit using second hyperbolic function. The pulse duration is defined as a full width half maximum (extracted from fit), divided by a factor of 1.53 for a Gaussian pulse profile

2 Pulse Energy and Intensity Dependence of Transient Absorption Spectrum

Figure S3a shows pump wavelength dependence of the transient absorption spectra, measured 2 ps after pump pulses. Pump fluences for all wavelengths were $\sim$4.33 mJ/cm^2. We observe the highest transient response when the pump energy is higher than the bandgap of $NiPS_3$ (1.8 eV). This shows that the transient response is dominated thermal heating of electronic and magnetic subsystems, which resulted form efficient generation of electron-hole pairs. In Figures S3b we show fluence dependence of magnetic exciton absorption peaks. Oscillator strengths of both peaks (I and II) go linearly with pump intensities. All the experiments we measured at 10K.

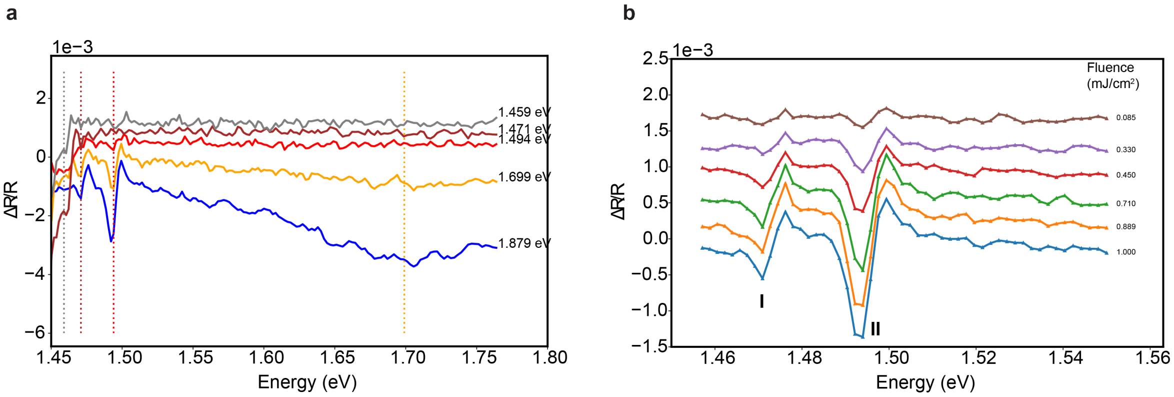

Figure S3: a. Pump energy dependence of the response. Only pumping above the optical bandgap gives high response and the oscillatory features start to become clear. **b.** Pump fluence dependence of the excitonic intensities. Both (**a**) and (**b**) were taken at 10K.

3 Time Resolved Absorption Spectrum

Time and energy resolved absorption spectrum trace is given in Figure S4. In this experimental modality, the pump fluence used is 4.33 mJ/cm^2. The high pump fluence bleaches the magnetic spectral response by quenching the magnetic order. As shown in the figure, the bleaching of magnetic excitons and d-d transitions persists up to 80 picoseconds. The observation of long term behaviour shows that these spectral features bleach due to steady state heating of $NiPS_3$.

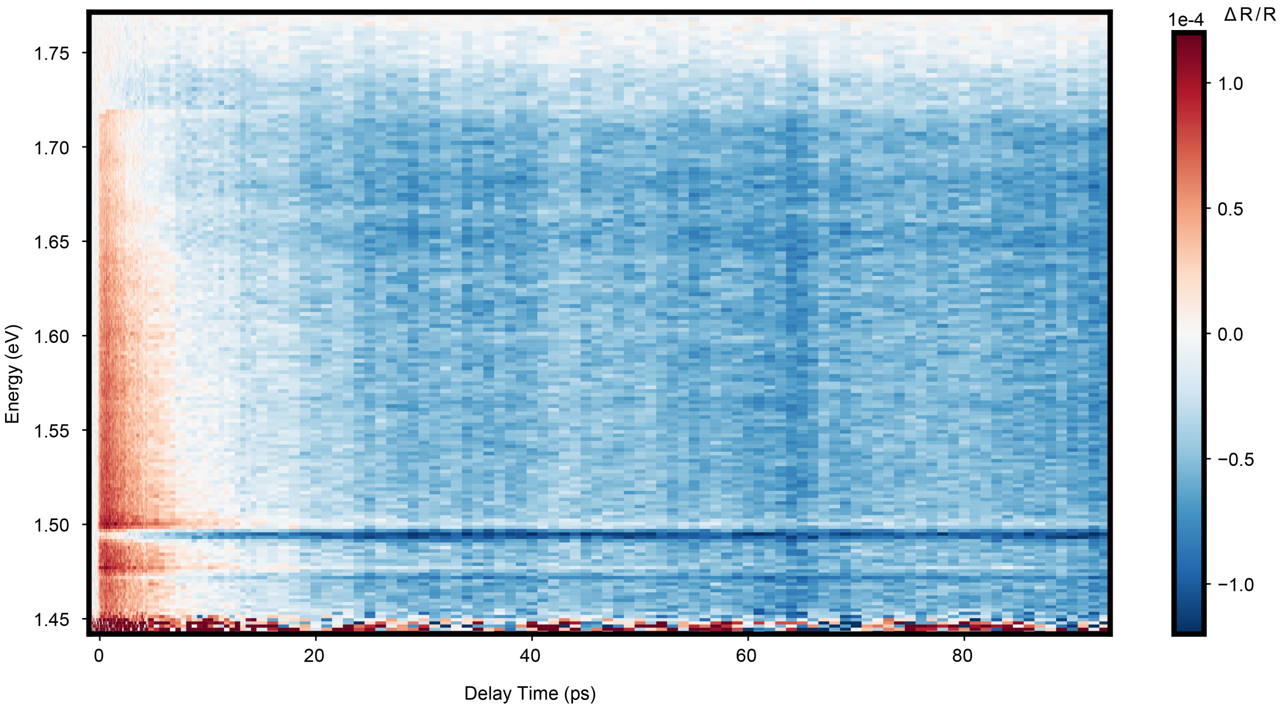

Figure S4: Time dependence of absorption. Two narrow lines in the lower side are peak I (exciton) and peak II. Due to their previously reported exceptionally sharp bandwidth, their lifetimes are at least 100 ps. Besides that the spectral oscillations also last for long (> 100 ps) after excitation. This shows that the spectral oscillations are indeed equilibrium phenomena. The data was taken at 30K.

4 Temperature Dependence of Replica Spacings

Figure S5 shows the temperature dependence of the Fourier spectrum of spectral oscillations. The oscillatory features near the d-d transition are extracted by fitting a smooth background to the d-d transition region. The temperature dependence of the Fourier modes show a broad peak around 28 meV, corresponding to the phonon mode contributing to the electron-phonon bound states. The center frequency of the Fourier component does not change as a function of temperature, and does not exhibit any softening. This observation rules out any scenario that invokes magnetic collective modes as the bosonic field responsible for replica formation.

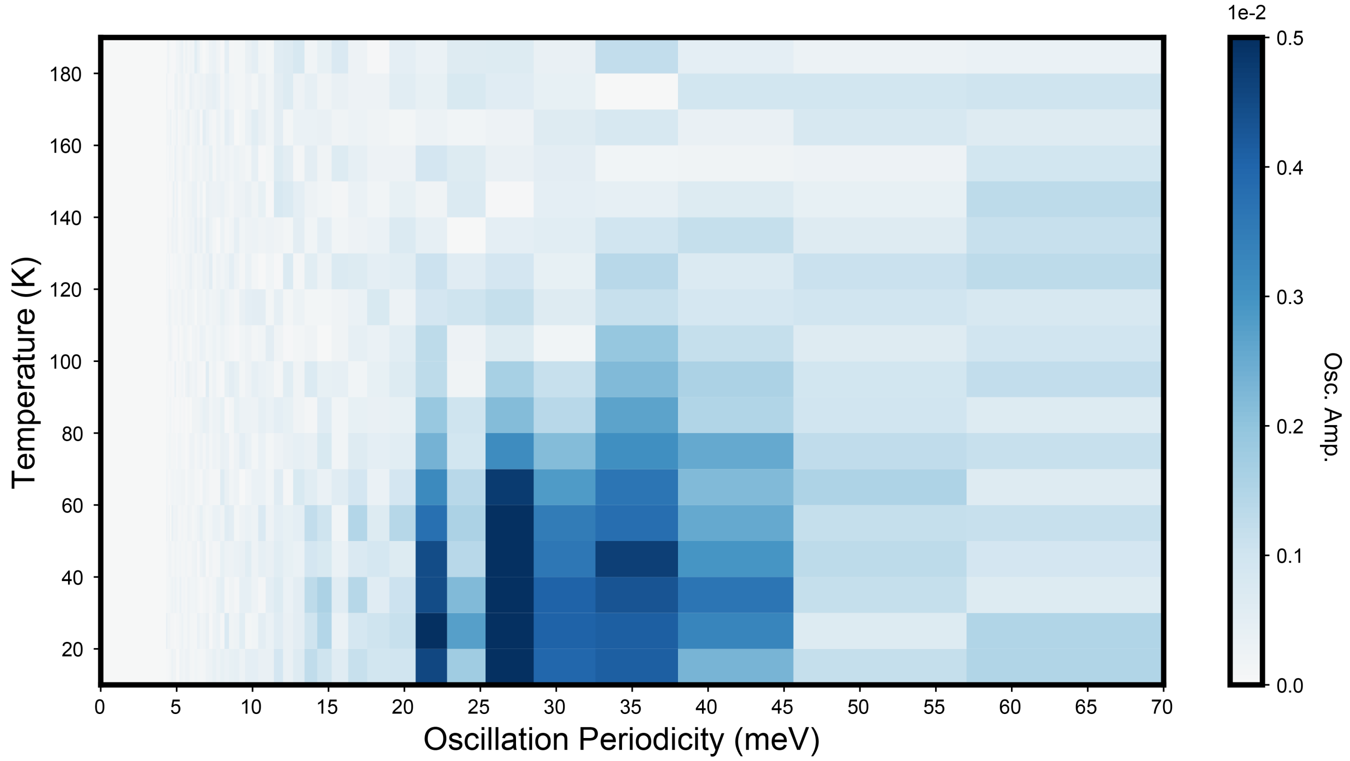

Figure S5: Temperature dependence of the Fourier modes of the spectral oscillations. No signs of mode softening was observed, as expected for phononic replicas.

5 Temperature Dependent Coherent Phonon Spectroscopy

Figure S6a shows temperature dependent traces obtained with energy integrated coherent phonon spectroscopy. In this experimental scheme, a low fluence level ($\sim$1 μJ/cm^2) is used not to perturb the magnetic order. The pulse duration used in this experiments is $\sim$25 fs, that coherently launch phonon modes via impulsive stimulated Raman scattering. To extract the transient response that corresponds coherent phonon oscillations, we fit the overall traces to an exponential background. The extracted oscillatory modes are shown below. Coherent phonon traces are dominated by the 7.5 THz A_{1g} phonon mode, and not show a clear temperature dependence. This indicates the 7.5 THz A_{1g} phonon is present both above and below the Neel temperature.

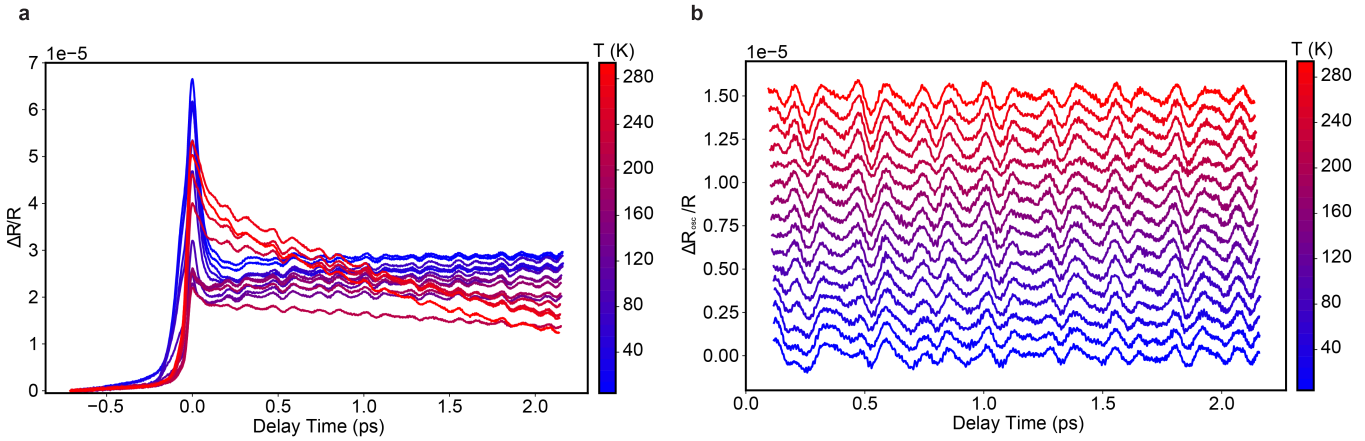

Figure S6: a. Temperature dependent transient reflectivity, with high temporal resolution ($\sim$ 25 fs). **b.** The oscilatory part of the pump-probe signal was extracted by subtracting the background with an exponential fit. No visible change in phonon properties (energy, phase or amplitude) were detected.

6 Energy Dependent Coherent Phonon Spectroscopy

In energy dependent coherent phonon spectroscopy, we send the probe beam into a monochromator after it gets reflected from the sample. Two dimensional energy dependent coherent phonon traces are obtained after subtracting the fitted incoherent response from the data at each wavelength. The 2D trace is given in Figure S7a. The Fourier transform of this 2D trace is given in Figure S7b, which shows that the dominant phonon response is at 7.5 THz and amplitude of it starts to increase in the d-d electronic transition region.

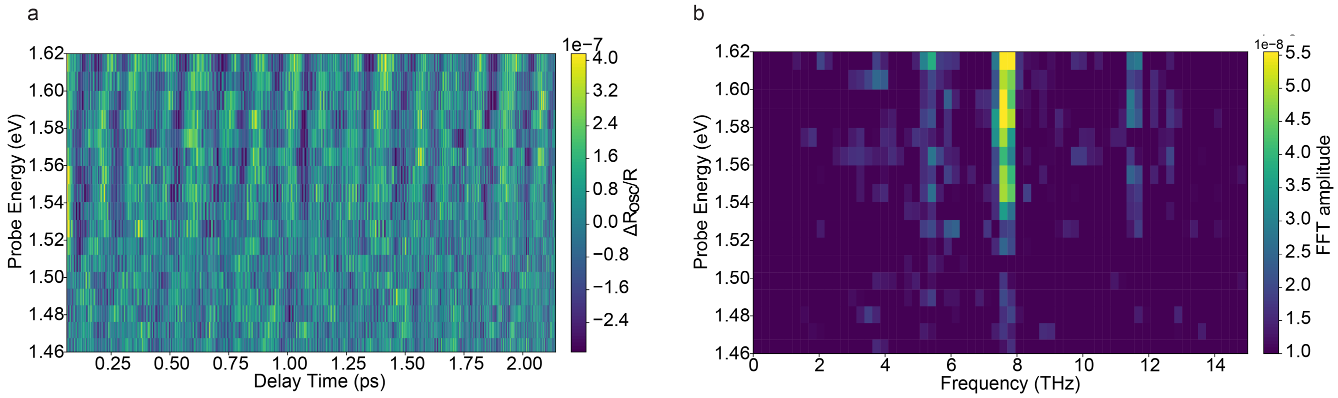

Figure S7: a. Spectrally resolved transient reflectivity data with high temporal resolution. It can be seen that the oscillations are more visible in the upper region, where the d-d level is located. **b.** Fourier transform of (**a**). Central bright line is the 7.5 THz, A_{1g} mode, amplitude of which decays near excitonic region (below 1.5 eV). Two other weaker lines are 5.2 THz and 11.5 THz modes.

7 Electronic transitions within d-levels

As mentioned in the main text, the d-d electronic transition from ${}^3A_{2g} \rightarrow {}^3T_{1g}$ is a spin allowed, parity forbidden single electron transition (see Figure S8a) and the corresponding level splitting is ∼1.7 eV. Another transition from ${}^3A_{2g} \rightarrow {}^3T_{2g}$ (1.1 eV) is also observed in a broadband absorption experiment [2] at low temperatures. Both of these transitions are parity forbidden, hence requires broken inversion symmetry in the system globally or locally near nickel sites [4]. As $NiPS_3$ is known to be centrosymmetric system (Figure S8b) [1], the visibility of the transitions among d-levels in the absorption spectrum, implies a locally broken inversion symmetry. Temperature dependent absorption spectra hints to magnetic origin of this local symmetry breaking, as was discussed in the main text.

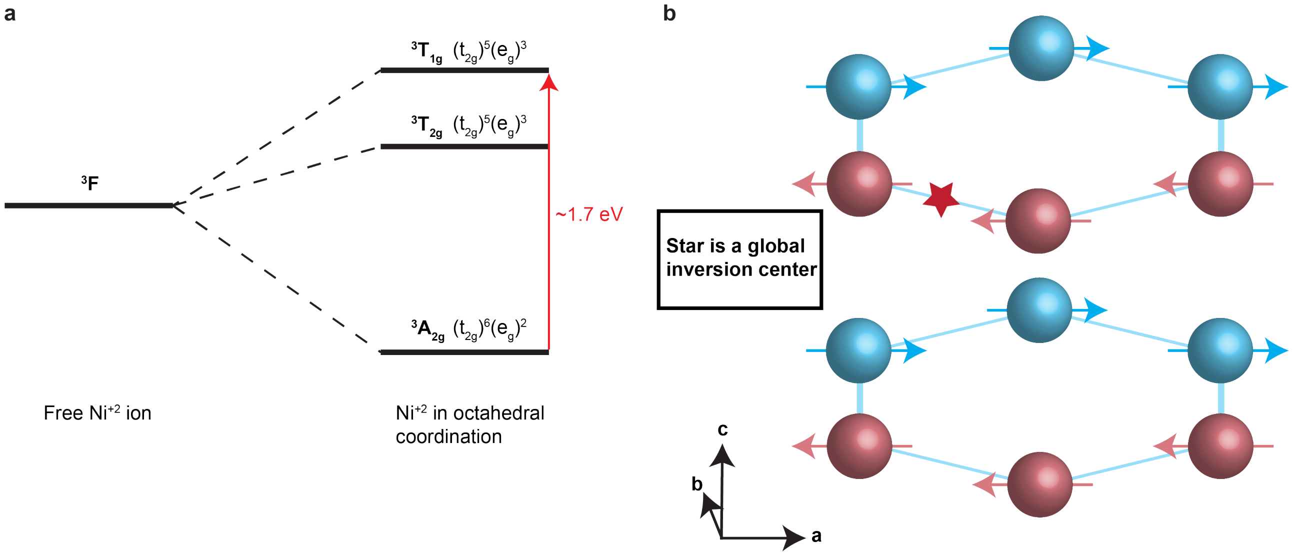

Figure S8: a. Energy levels scheme of Ni^{+2} ion in octahedral coordination and when it is a free ion. The ground state of Ni^{+2} in the octahedral coordination is ${}^3A_{2g}$ triplet, with 6 electrons in the lower lying t_{2g} level and 2 electrons in the the upper e_g level. The absorption band of our interest is indicated with a red arrow, corresponds to ${}^3A_{2g} \rightarrow {}^3T_{1g}$ single electron transition, which is spin allowed and electric dipole forbidden. **b.** Magnetic structure of $NiPS_3$. All the spheres (blue and red) are nickel ions. Arrows indicate the spin vector and a red star is an inversion center of a crystal. Figure is motivated by Chu et al[1].

8 Difference between broadband transient absorption spectroscopy and coherent phonon spectroscopy

In our study, we use two different ultrafast schemes to measure magnetically enabled electron-phonon bound states. Despite the similarities of optical techniques used, both of these experiments measure different observables.

Within our minimal model described in the main text, we can use Fermi's golden rule to describe the equilibrium absorption of $NiPS_3$ with the following equation:

$$\text{Abs}(\omega) = \frac{4\pi^2}{\hbar^2} d(T) e^{-g} \sum_{n=0}^{\infty} \frac{g^n}{n!} L(\omega - \omega_{dd} + \Delta - \omega_{ph} n) \tag{1}$$

where ω_{dd} describes the energy of d-d electronic transition, ω_{ph} is the phonon frequency, g is called a dimensionless Huang-Rhys factor and L is a Gaussian function corresponds to the lineshape of undressed d-d transition. In this expression, $d(T)$ is the temperature dependent dipole matrix element that vanishes for an d-d level in an inversion symmetric potential landscape. Therefore, without the local inversion symmetry breaking contribution from the magnetic order, the absorption around the d-d transition is zero. Below the magnetic ordering temperature of $NiPS_3$, the dipole matrix element becomes non-zero and is a function to the temperature.

In our broadband transient absorption spectroscopy measurements, an intense pump pulse with a pulse duration of 200 fs quenches the magnetic order without coherently launching any phonon modes. Assuming that the pump pulse increases the sample temperature from T_i to T_f, we can write the transient change in reflectivity in the following form:

$$\Delta\text{Abs}(\omega) = \frac{4\pi^2}{\hbar^2} (d(T_i) - d(T_f)) e^{-g} \sum_{n=0}^{\infty} \frac{g^n}{n!} L(\omega - \omega_{dd} + \Delta - \omega_{ph} n) \tag{2}$$

In the case of final temperature exceeding the magnetic temperature, the broadband transient absorption spectroscopy is proportional to the equilibrium absorption.

Despite the similarities in experimental setups, energy resolved coherent phonon spectroscopy measures a different observable. Compared to the fluence levels used in broadband transient absorption spectroscopy ($\sim$ 4 mJ/cm^2), coherent phonon spectroscopy uses much lower fluence levels ($\sim$ 1 μJ/cm^2) and shorter pulses. This allows us to excite phonons coherently while keeping the magnetic subsystem intact. Therefore, we can approximate the quantum state after the excitation in the following way:

$$|\Psi(t)\rangle = |\Psi_{\text{electronic}}\rangle \otimes |\alpha_{\text{phonon}}\rangle \tag{3}$$

where $|\alpha_{\text{phonon}}\rangle$ is the coherent phonon quantum state and $|\Psi_{\text{electronic}}\rangle$ is the electronic and magnetic system. In this approximation, the Hamiltonian used in the main text can be simplified into the following form:

$$H_{el} = \hbar(\omega_{dd} + M\alpha \cos(\omega_{ph} t))\hat{\sigma}^\dagger \hat{\sigma} \tag{4}$$

This effective Hamiltonian represents a two level system, the energy of which changes in time. This implies that any two level system that couples to the phonons in the following form will undergo a change in energy and this should be reflected in the coherent phonon traces. Since magnetic excitons do not show any signatures of phonon mode dependent energy shift, we can suggest that they are not coupled to the 7.5 THz A_{1g} phonon modes.

In addition to this coupling, coherent excitations of Raman active modes will modulate the macroscopic polarizability, which is proportional to the dipole matrix element. Frequency dependent changes in polarizability ($\alpha(\omega)$) due to a coherently launched phonon can be written as:

$$\alpha(\omega) = \alpha_0(\omega) + \frac{\partial \alpha(\omega)}{\partial Q} Q \cos(\omega_{ph} t) \tag{5}$$

In this case, the energy resolved coherent phonon spectroscopy measures the $\frac{\partial \alpha(\omega)}{\partial Q}$ as a function of frequency. In the spectral region corresponding to the magnetic excitons, this expression is proportional to the changes in its dipole matrix element as a function of phonon displacement. Therefore, with our experimental observations, we can conclude that the phonons do not modulate the dipole matrix element of the magnetic excitons, implying a negligable coupling between them.

9 Electron-phonon coupling

As we describe in the main text, the effective Hamiltonian (Eq. 1) captures the interaction between localized d-electron of nickel ions and phonons. The Hamiltonian can be exactly diagonalized for this model [3], and its spectral function is given by:

$$A(\omega) = 2\pi e^{-g} \sum_{n=0}^{\infty} \frac{g^n}{n!} L(\omega - \omega_{dd} + \Delta - \omega_{ph} n) \tag{S.1}$$

where L function corresponds to the lineshape of undressed d-d transition, ω_{ph} is a phonon frequency, ω_{dd} is a frequency corresponding to d-d transition and Δ is a renormalization energy of d-d transition due to coupling to phonons, which is equal to $g\omega_{ph}$. In this function, g is the Huang-Rhys factor which is related to dimensionless electron phonon coupling.

We first extract the parameters of our minimal by approximating the lineshape of undressed d-d transition as Gaussian. Then, the functional form given above corresponds to a Gaussian lineshape that is convolved with a Poissonian distribution. The fit to data is shown in Fig. 4 and extracted fit parameters are given below:

g	ω_{ph} (meV)	ω_{dd} (eV)	σ (meV)
10.046 ± 0.058	27.7 ± 0.38	1.735 ± 0.002	11.5 ± 0.2

We then extract the parameters of our minimal by approximating the lineshape of undressed d-d transition as Lorentzian. Then, the functional form given above corresponds to a Lorentzian lineshape that is convolved with a Poissonian distribution. The fit to data is shown in Fig. S9 and extracted fit parameters are given below:

g	ω_{ph} (meV)	ω_{dd} (eV)	FWHM (meV)
9.919 ± 0.089	29.6 ± 0.49	1.735 ± 0.004	29.2 ± 0.5

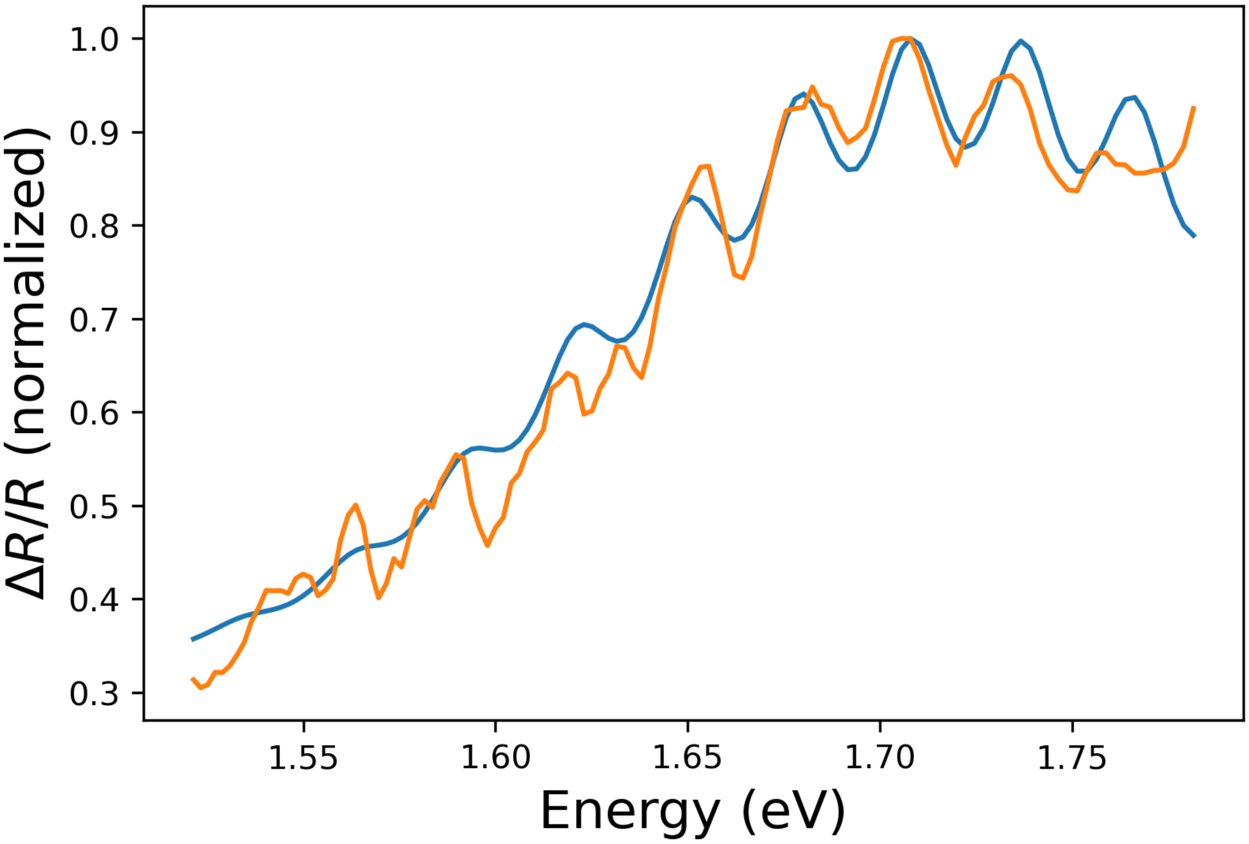

Figure S9: Fit to the transient absorption spectroscopy using Lorentzian lineshape

10 Difference between coherent phonon spectroscopy and Raman scattering

Although both Raman and time resolved coherent phonon spectroscopy measure the zone center phonon modes, each of these techniques excite and detect the phonon modes differently.

In the case of Raman spectroscopy of phonon modes, a narrow band laser interacts with the phonon modes and scatters off of the sample inelastically. At zero temperature, this inelastic process causes a transition from zero phonon ground state and electronic ground state $|g_{\text{electronic}}, 0_{\text{phonon}}\rangle$ to a single phonon excited and electronic ground state. In this process, the light frequency redshifts by the phonon energy. The cross section of this spontaneous Raman process for a particular phonon mode is given in the following form:

$$\sigma_{\text{Raman}}(\omega_{\text{excitation}}) = \sum_e \frac{\langle g_{\text{electronic}}, 1_{\text{phonon}}| x |e\rangle \langle e| x |g_{\text{electronic}}, 0_{\text{phonon}}\rangle}{\omega - \omega_e + i\Gamma} \tag{S.2}$$

where x is the position operator, $\omega_{\text{excitation}}$ refers to the incident laser frequency and e refers to every possible excited state in this system in equilibrium.

Raman spectroscopy of phonon modes is an equilibrium technique to determine the position of phonon modes, as the excitation laser do not cause any strong change in electronic or magnetic structure other than steady state heating. In addition, as shown in equation S.2., the Raman cross section is highly sensitive to the excited state structure. Any temperature dependent change of excited spectrum can result in a change in Raman peak intensities.

On the other hand, coherent phonon spectroscopy observes the phonon modes in real time by measuring the change in reflectivity in real time following a pump excitation. The energy integrated differential reflectivity without any energy resolution as a function of pump-probe pulse delay can be depicted in the following form:

$$(\frac{\Delta R(\tau)}{R})_{\text{integrated}} = \frac{\int \Delta R(\tau, \omega) A(\omega) d\omega}{\int R(\omega) A(\omega) d\omega} \tag{S.3}$$

where $A(\omega)$ denotes the laser spectrum and $\Delta R(\tau, \omega)$ is the energy resolved differential reflectivity.

The energy resolved differential reflectivity can be decomposed into the incoherent part pertaining to excited electron decay dynamics and the oscillatory part due to phonon excitations. The oscillatory part of the energy integrated differential reflectivity can be expressed as:

$$(\frac{\Delta R(\tau)}{R})_{\text{osc}} = \sum_i \Delta Q_i(\tau) \frac{\int \frac{\partial R(\omega)}{\partial Q_i} A(\omega) d\omega}{\int R(\omega) A(\omega) d\omega} \tag{S.3}$$

where ΔQ_i is pump induced, displacively or impulsively, phonon displacement. The sum runs over all phonon modes that are excited. For a specific phonon mode, the phonon amplitude in reflectivity is proportional to:

$$(\Delta R(\tau))_{\text{phonon}} = \Delta Q_i(\tau) \int \frac{\partial R(\omega)}{\partial Q_i} A(\omega) d\omega \tag{S.3}$$

In our experiments, the pump and probe pulses span a broad spectral window starting from 1.5 eV to 1.9 eV. In $NiPS_3$, this spectral range covers the magnetic excitons, d-d transitions and charge transfer gap. Since magnetic excitons do not couple to the phonon modes of interest, the energy integrated coherent phonon spectroscopy measures the phonon modulation of reflectivity in the spectral region involving d-d transitions and charge transfer gap.

Even though Raman and coherent phonon spectroscopy couple to phonon modes, the expressions governing their amplitudes are distinct from each other and can have different temperature dependences.

References

[1] Hao Chu et al. "Linear Magnetoelectric Phase in Ultrathin MnPS3 Probed by Optical Second Harmonic Generation". In: *Phys. Rev. Lett.* 124 (2 2020), p. 027601. DOI: 10.1103/PhysRevLett.124.027601. URL: https://link.aps.org/doi/10.1103/PhysRevLett.124.027601.

[2] So Yeun Kim et al. "Charge-Spin Correlation in van der Waals Antiferromagnet NiPS3". In: *Physical Review Letters* 120.13 (2018), p. 136402. ISSN: 10797114. DOI: 10.1103/PhysRevLett.120.136402.

[3] Gerald D. Mahan. *Many-Particle Physics.* Springer, 2000. DOI: 10.1007/978-1-4757-5714-9.

[4] Xiuwen Zhang et al. "Hidden spin polarization in inversion-symmetric bulk crystals". In: *Nature Physics* 10.5 (2014), pp. 387–393. ISSN: 17452481. DOI: 10.1038/nphys2933.
